# Successful Treatment of Neutropenia Associated With T‐Cell Large Granular Lymphocytic Leukemia Using Fludarabine

**DOI:** 10.1155/crh/4086081

**Published:** 2026-01-20

**Authors:** Yoshiki Uemura, Kazuto Togitani, Yoshitaka Kumon

**Affiliations:** ^1^ Department of Hematology, Chikamori Hospital, 1-1-16 Okawasuzi Kochi-shi, Kochi, 780-8522, Japan, chikamori.com; ^2^ Department of Rheumatology, Chikamori Hospital, 1-1-16 Okawasuzi Kochi-shi, Kochi, 780-8522, Japan, chikamori.com

**Keywords:** case report, fludarabine, neutropenia, pegfilgrastim, T-cell large granular lymphocytic leukemia

## Abstract

T‐cell large granular lymphocytic leukemia (T‐LGLL) is an uncommon lymphoproliferative disorder that typically follows a slow clinical course. Symptoms often remain subtle until cytopenia or infection develops. Severe infection secondary to neutropenia represents the major cause of mortality. We describe an uncommon case involving an 81‐year‐old woman diagnosed with T‐LGLL whose agranulocytosis was followed by recurrent infections. Immunosuppressive agents such as methotrexate, cyclophosphamide, and tacrolimus—commonly recommended by current guidelines—were administered but failed to improve neutropenia. Administration of fludarabine, a purine analog listed as a second‐line option in the NCCN guidelines, led to a prompt rise in neutrophil counts and a concomitant decline in LGL levels. To our knowledge, no prior Japanese report has documented successful use of fludarabine monotherapy for T‐LGLL‐related neutropenia, prompting us to describe this case.

## 1. Introduction

Felty syndrome (FS) is a rare complication of rheumatoid arthritis (RA), defined by the triad of splenomegaly, neutropenia, and joint destruction, occurring in fewer than 1% of RA patients [1]. Peripheral expansion of large granular lymphocytes (LGLs) occurs in roughly one‐third of FS cases [2]. Clonal proliferation of cytotoxic T‐cell–type LGLs defines T‐cell large granular lymphocytic leukemia (T‐LGLL) [3]. T‐LGLL frequently coexists with autoimmune disorders, including RA, FS, Coombs‐negative hemolytic anemia, immune thrombocytopenia, and pure red cell aplasia [4–6]. RA occurs in approximately 11%–36% of patients with LGLL [7]. Given their overlapping clinical and immunologic profiles, FS and T‐LGLL are considered possible phenotypic variants within the same pathogenic continuum [8].

Distinction between FS and T‐LGLL often relies on the presence of T‐cell receptor (TCR) gene rearrangements [9]. Our patient was diagnosed with T‐LGLL associated with RA based on confirmation of TCR gene rearrangement. Because T‐LGLL typically progresses slowly, observation without immediate treatment is reasonable in asymptomatic cases [10]. Treatment is indicated when cytopenias, infections, progressive splenomegaly, or systemic “B” symptoms develop [11].

Since leukemic LGLs function as persistently activated cytotoxic lymphocytes, immunosuppression remains the cornerstone of therapy [3, 12, 13]. First‐line options include methotrexate (MTX), cyclophosphamide (CPA), or cyclosporine (CyA), administered singly [14, 15]. MTX often benefits patients with neutropenia, whereas CPA is more effective for anemia, particularly pure red cell aplasia [16, 17]. A clinical response typically requires several months of continuous therapy [16]. Because prolonged immunosuppression can increase infection risk, therapies capable of rapidly restoring neutrophils are desirable.

## 2. Case Presentation

An 81‐year‐old woman had been diagnosed with RA in 2012 and was referred to our hospital in September 2020 for active arthritis and agranulocytosis. Because arthritis activity was high, so mizoribine, tacrolimus, and salazosulfapyridine were started (Figure 1(a)). Because of coexisting RA and neutropenia, FS was suspected, though splenomegaly was absent. As no infectious events were seen, she was observed without specific treatment for neutropenia.

Figure 1Clinical parameters and treatments from December 2022 to September 2025. (a) Clinical course from the first visit to near‐zero neutrophil count (February 5, 2021–December 20, 2022). MZB, mizoribine; TAC, tacrolimus hydrate; SASP, salazosulfapyridine; WBC, white blood cell; Hb, hemoglobin; PLT, platelet; Neut, neutrophil. (b) After febrile neutropenia (FN) treatment, neutrophils temporarily increased but later declined, while WBC showed an upward trend (December 20, 2022–May 7, 2024). FN treatment was initiated with meropenem (MEPM) but fever persisted; therefore, ceftriaxone (CTRX) and vancomycin (VCM) were added. As these were ineffective, cefepime (CFPM) combined with lenograstim led to defervescence and an increase in neutrophil count. ABT, abatacept; LGL, large granular lymphocyte; PSL, prednisolone; CFPM, cefepime; MEPM, meropenem; CTRX, ceftriaxone; VCM, vancomycin; LENO, lenograstim. (c) Immunosuppressive therapy was ineffective for severe neutropenia and increased LGL count. Combination therapy with fludarabine and pegfilgrastim (May 21, 2024–September 25, 2025) improved both parameters. RIT, rituximab; MTX, methotrexate; CPA, cyclophosphamide; FIL, filgrastim; PEGFIL, pegfilgrastim; Flu, fludarabine; MCFG, micafungin.

**Figure:**
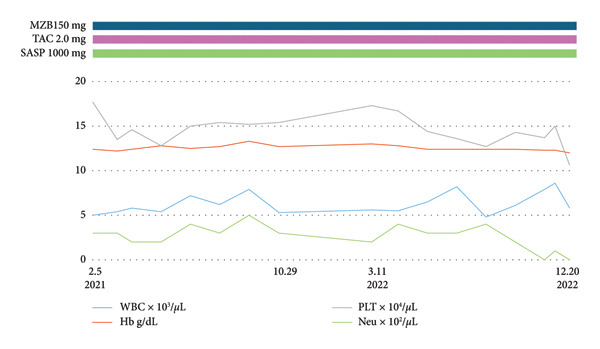
(a)

**Figure:**
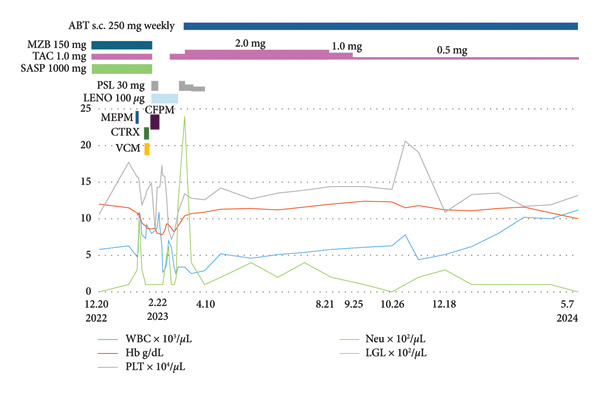
(b)

**Figure:**
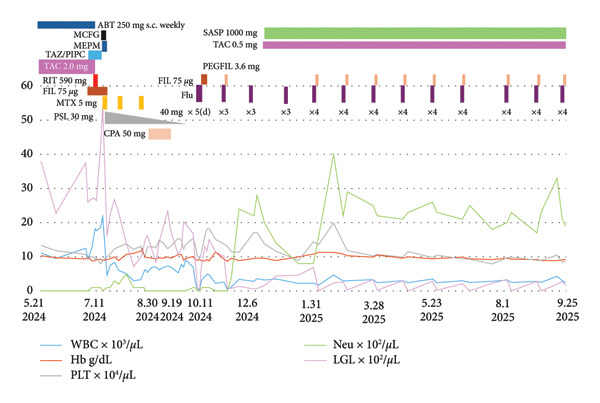
(c)

**Figure 2:**
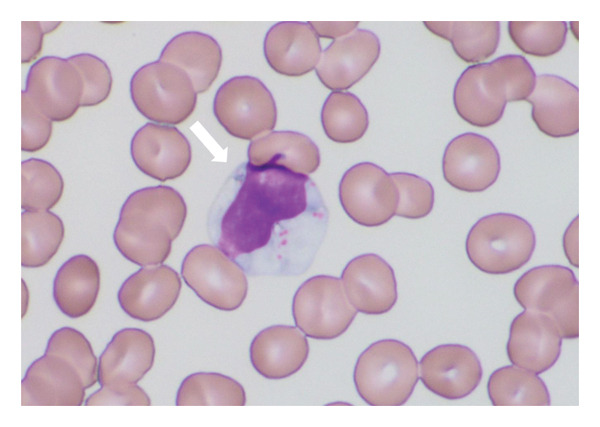
May–Grünwald–Giemsa staining of peripheral blood (× 1000). A white arrow indicates a large granular lymphocyte.

By November 2022, her neutrophil count had declined to < 100/μL. In February 2023, febrile neutropenia (FN) developed and was treated with antibiotics and filgrastim, resulting in transient recovery and fever resolution. To avoid recurrence, mizoribine and salazosulfapyridine were discontinued, and abatacept was introduced. Nevertheless, neutropenia worsened again in December 2023, while total WBC gradually increased (Figure 1(b)). By May 2024, WBCs were 11,200/μL, but neutrophils remained absent. Peripheral smear showed LGLs (34% of WBCs) (Figure 2). Flow cytometric analysis of peripheral lymphocytes revealed a CD2+CD3+CD5+CD7+CD8+CD57+ T‐cell population, consistent with cytotoxic T‐cell proliferation. TCR gene rearrangement analysis demonstrated clonality in both the TCR‐γ and TCR‐β chains, confirming the diagnosis of T‐LGLL. The laboratory findings at diagnosis are summarized in Tables 1 and 2)

**Table 1:** Clinical laboratory data and reference ranges of the patient at the time of T‐LGLL diagnosis.

Category	Parameter	Value	Reference range
Complete blood count	WBC	9600/μL	3300–8600/μL
	Seg	87%	45–60%
	Lymphocyte	9%	25–45%
	Mono	4%	4–7%
	RBC	154 × 10^4^/μL	386–492 × 10^4^/μL
	Hb	6.8 g/dL	11.6–14.8 g/dL
	Ht	20.1%	35.1–44.4%
	MCV	132.2 fL	83.6–98.2 fL
	PLT	9.4 × 10^4^/μL	15.8–34.8 × 10^4^/μL

Urinalysis	Color	yellow	—
	Protein	(+/−)	(−)
	Glucose	(−)	(−)
	Occult blood	(+)	(−)

Urinary sediments	RBC	1–4/H	—
	WBC	30–40/H	—

Serological test	Haptoglobin	87 mg/dL	—
	Intrinsic factor Ab	(+)	(−)

Genetic test	WT1 mRNA	2.3 × 10^2^ copy/μg RNA	< 50 copy/μg RNA

**Table 2:** Biochemical and bone marrow findings of the patient at the time of T‐LGLL diagnosis

Category	Parameter	Value	Reference range
Biochemistry tests	TP	5.6 g/dL	6.6–8.1 g/dL
	Alb	3.5 g/dL	4.1–5.1 g/dL
	AST	22 U/L	13–30 U/L
	ALT	15 U/L	7–23 U/L
	ALP	386 U/L	38–113 U/L
	γ‐GTP	219 IU/L	9–32 U/L
	CHE	13 IU/L	240–486 U/L
	LDH	378 U/L	124–222 U/L
	T‐bil	1.0 mg/dL	0.4–1.5 mg/dL
	BUN	19.8 mg/dL	8–20 mg/dL
	Cr	0.42 mg/dL	0.46–0.79 mg/dL
	UA	3.4 mg/dL	2.6–5.5 mg/dL
	T‐CHO	137 mg/dL	142–248 mg/dL
	TG	86 mg/dL	40–234 mg/dL
	Glu	142 mg/dL	73–109 mg/dL
	HbA1c	4.80%	4.3–5.8%
	Na	137 mEq/L	138–145 mEq/L
	K	4.5 mEq/L	3.6–4.8 mEq/L
	Cl	103 mEq/L	101–108 mEq/L
	CRP	0.66 mg/dL	0.00–0.14 mg/dL
	Fe	44 μg/dL	80–200 μg/dL
	UIBC	126 μg/dL	168–252 μg/dL
	Ferritin	439.4 ng/mL	39.4–340 ng/mL
	Vitamin B12	57 pg/mL	180–914 pg/mL
	Folic acid	2.1 ng/mL	3–20 ng/mL

Bone marrow findings	NCC	34 × 10^4^/μL	
	Mgk	345/μL	
	Pro‐E	0.2%	
	Ba‐E	4.4%	
	Poly‐E	19.8%	
	Orth‐E	1.0%	
	Myeloblast	0.6%	
	Pro‐mye	2.0%	
	Mye	13.4%	
	Meta‐Myelo	9.8%	
	Stab	9.8%	
	Seg	30.2%	
	Eosino	1.0%	
	Baso	0.2%	
	Lymphocyte	0.4%	
	Mono	0.4%	
	Plasma	2.2%	
	MΦ	0.6%	
	M/E	2.7	
	Chromosome	46, XX	

A recurrent FN episode in July 2024 required broad‐spectrum antibiotics. Rituximab on July 11 plus filgrastim achieved minimal response [18]. MTX (5 mg/week) was started on July 19, 2024, with PSL 30 mg/day (tapered thereafter); due to persistent neutropenia, MTX was stopped on August 23 and CPA 50 mg/day was given from August 30 to September 19, 2024, with only incidental overlap from PSL tapering—not intentional combination therapy. Because alemtuzumab was unavailable in Japan, oral fludarabine was selected per NCCN guidance. Treatment began on October 11, 2024. The first course consisted of (40 mg/day x 5 days per month) was followed by filgrastim for 8 days. During the second and third courses (40 mg/day x 3 days, no G‐CSF), transient neutropenia and LGL resurgence occurred. From the fourth course onward, fludaraine (40 mg/day x 4 days) plus pegfilgrastim after each cycle lead to sustained neutrophil recovery and continuous LGLs decline. This response persisted throughout the observation period until September 25, 2025 (Figure 1(c)). Because of arthritis flare, salazosulfapyridine and tacrolimus were reintroduced. The overall course is summarized in Figure 1(c).

## 3. Discussion

FS is defined by the coexistence of RA, splenomegaly, and neutropenia [19]. It usually emerges > 10 years after RA onset [20]. In our patient, severe neutropenia appeared over 10 years after RA diagnosis but without splenomegaly, so FS was initially suspected. LGLL is not always indolent; rather, the disease frequently has an indolent course. Approximately 85% are T‐cell type [21].

RA is present in 11%–36% of T‐LGLL patients [7, 22], whereas clonal LGL expansion occurs in 5%–10% of RA patients, indicating a bidirectional association and shared pathogenesis [8, 23]. Our patient presented with RA and persistent agranulocytosis; TCR gene rearrangement confirmed T‐LGLL, demonstrating this overlap.

Given its indolent nature, T‐LGLL is often observed without therapy, but treatment is warranted for severe cytopenia or infection. Because RA, FS, and T‐LGLL are considered to lie on a pathogenic continuum, we initially attempted to suppress RA activity using rituximab, hoping this would attenuate LGL‐driven neutropenia. Although previous reports describe improvement of cytopenias in RA‐associated LGL disorders following rituximab therapy [18], our patient showed only minimal hematologic response. Therefore, we proceeded with conventional immunosuppressive agents. First‐line immunosuppressants (MTX, CPA, and CyA) remain standard [7, 12–15]. In this case, MTX plus prednisolone followed by CPA were ineffective, underscoring the limitation of conventional therapy.

Although methotrexate and cyclophosphamide usually require several weeks to months for an adequate assessment of efficacy in T‐LGLL, our patient had profound agranulocytosis and recurrent infections, making prolonged treatment under severe neutropenia clinically unsafe. Neither MTX nor CPA produced any early indication of hematologic improvement, which justified the decision to discontinue these agents earlier than the conventional treatment duration.

Fludarabine, cladribine, and pentostatin are NCCN category 2A options for refractory T‐LGLL [7]. Fludarabine inhibits DNA synthesis in proliferating lymphocytes and may explain the rapid neutrophil recovery observed here. Although previous reports showed variable responses [24–26], our case suggests that combining fludarabine with pegfilgrastim may accelerate hematologic recovery without LGL re‐expansion.

While a durable response was achieved, long‐term safety in elderly patients with autoimmune backgrounds remains uncertain. Because the observation period in this case remains relatively short, longer follow‐up is required to determine whether hematologic remission and clinical stability can be maintained over the long term.

In summary, this case highlights that oral fludarabine combined with pegfilgrastim can induce rapid and sustained neutrophil recovery in T‐LGLL refractory to standard immunosuppressive therapy, providing a feasible outpatient option for elderly patients with RA‐associated disease.

## Ethics Statement

Ethical approval was waived by the institutional review board of Chikamori Hospital because this is a single‐patient case report that does not contain identifiable patient information.

## Consent

Written informed consent was obtained from the patient for publication of this case report and any accompanying images.

## Disclosure

All authors have read and approved the final version of the manuscript.

## Conflicts of Interest

The authors declare no conflicts of interest.

## Author Contributions

Yoshiki Uemura contributed to the conception, design, and drafting of the manuscript. Kazuto Togitani supervised the clinical management and critically reviewed the manuscript. Yoshitaka Kumon contributed to the diagnosis and management of rheumatoid arthritis and provided rheumatologic expertise. Yoshiki Uemura had full access to all of the data in this study and takes complete responsibility for the integrity of the data and the accuracy of the data analysis.

## Funding

No specific grant or funding was received from any public, commercial, or not‐for‐profit sector for this study.

## Data Availability

All data supporting the findings of this case are available in the patient’s medical records at Chikamori Hospital, Kochi, Japan. Further details are available from the corresponding author upon reasonable request, with appropriate ethical approval.
